# Molecular Dynamics Simulation of the Interfacial Characteristics of Functionalized Carbon Nanotube-Polyimide Composites

**DOI:** 10.3390/polym18131673

**Published:** 2026-07-06

**Authors:** Youyun Zou, Yi Liu, Xin Zha, Ang Wang, Zongrong Wang, Jin Qian

**Affiliations:** 1 Center for X-Mechanics, Key Laboratory of Soft Machines and Smart Devices of Zhejiang Province, School of Aeronautics and Astronautics, Zhejiang University, Hangzhou 310027, China; youyunzou@zju.edu.cn (Y.Z.); zhaxin@zju.edu.cn (X.Z.); 2 State Key Laboratory of Silicon and Advanced Semiconductor Materials, School of Materials Science and Engineering, Zhejiang University, Hangzhou 310058, China; yi_liu@zju.edu.cn; 3 Institute of Thermal Science and Technology, Shandong University, Jinan 250061, China; 202334306@mail.sdu.edu.cn

**Keywords:** carbon nanotubes, polyimide, molecular dynamics, interfacial interaction

## Abstract

Insufficient interfacial interaction between nanoconductive materials and polymer matrices severely limits the mechanical, electrical, and pressure-sensing properties. Carbon nanotubes (CNTs), widely used as polymer reinforcements due to their excellent properties, can significantly enhance the mechanical performance of nanocomposites by improving the interfacial interactions with the matrix. Given the diversity of functionalized CNTs, a systematic study of their interfacial bonding mechanisms is of great importance for both scientific research and engineering applications. To this end, this study employs molecular dynamics simulations to investigate the interfacial characteristics and mechanical responses of functionalized CNT/polyimide (PI) systems. The results demonstrate that functionalization treatments significantly enhance both the interfacial interaction and the shear performance of CNT/PI nanocomposites. Specifically, the interfacial shear strength of the carboxylated CNT/PI composite reaches 269.83 MPa, representing a 20% improvement; furthermore, this property further increases with higher functional group content. This work elucidates the influence of functional group type and content on the interfacial shear performance of CNT/PI composites at the atomic scale, providing new physical insights.

## 1. Introduction

Due to their flexibility, high sensitivity, lightweight nature, and other advantageous properties, flexible pressure sensors have found extensive applications in fields such as health monitoring [1,2], electronic skin (e-skin) [3,4], and energy security [5]. Compared to other types of sensors, piezoresistive sensors have emerged as a highly competitive sensing technology, primarily due to their higher sensitivity, wider sensing range, and superior stability [6,7]. Commonly utilized conductive fillers for such sensors include carbon nanotubes (CNTs) [8], graphene [9], and metallic nanomaterials [10]. The dispersion uniformity and distribution density of conductive fillers are critical factors determining sensor performance, as they influence the formation of the conductive network and the stress-to-resistance conversion efficiency [11,12,13]. Hyang et al. [14] introduced multi-walled CNTs (MWCNTs) and carboxyl-functionalized MWCNTs (MWNT-COOH) into polyimide (PI), demonstrating that strong interfacial interaction substantially improved both dispersibility and interfacial adhesion. CNTs, due to their high aspect ratio and unique one-dimensional tubular structure, facilitate the formation of efficient conductive networks in polymer matrices. To enhance CNT dispersibility, numerous studies [15,16,17,18] have investigated CNT surface modification effects on thermomechanical properties of polymer composites. However, the interfacial mechanical properties between functionalized CNTs and polymer matrices remain unclear [19,20].

However, evaluating the interfacial mechanical properties of nanocomposites through experimental methods presents significant complexities and limitations [21]. For instance, understanding how functional groups affect interfacial properties during load transfer may require analysis at the mesoscopic or even microscopic scale to study real-time interactions between atoms of the two systems [22,23,24]. Consequently, many researchers have increasingly preferred using molecular dynamics (MD) simulations to investigate the interfacial mechanical properties [25,26,27,28] between polymer matrices and functionalized CNT. MD simulations enable nanoscale modeling of interfacial phenomena and have been widely applied to study interfacial behaviors in various material systems [29,30,31]. Jiang et al. [32] have used MD to predict changes in matrix order after CNT reinforcement, demonstrating that at the polymer–CNT interface, the degree of order increases with the volume fraction of MWCNTs. By simulating the constant-force pull-out process of CNTs from PI using MD [33], it was calculated that the ultimate pull-out force of three-walled CNTs is higher than that of single-walled carbon nanotube (SWCNT). The interfacial shear strength (ISS) of nanocomposites was also calculated based on pull-out energy, revealing that increasing the number of walls is a key factor in enhancing interfacial stress transfer during tension.

Although numerous studies have reported the interfacial mechanical properties between polymers and CNTs using MD simulation methods, there remains a lack of simulation research on the interfacial mechanical properties of functionalized CNT/PI nanocomposites. This study aims to investigate the effects of CNT functionalization on the interfacial and mechanical properties of PI nanocomposites and to understand how functionalization contributes to the modification of the CNT/PI interface at the atomic scale.

## 2. Simulation Details

### 2.1. Atomistic Models

This study systematically investigates the interfacial mechanical properties of pristine and functionalized CNT in a polyimide matrix. MD simulations were performed using the Large-scale Atomic/Molecular Massively Parallel Simulator (LAMMPS, 2 August 2023 release). The Polymer Consistent Force Field (PCFF) was employed to accurately describe the atomistic interactions within the nanocomposite systems. The validity of the PCFF for simulating CNT-reinforced nanocomposites has been well established in previous studies, which successfully captured the π-π bonding interactions between the aromatic rings of polymers and the surfaces of carbon materials. In this study, the interfacial bonding strength is primarily characterized by van der Waals (vdw) interactions and Coulomb forces between charged species. Specifically, Lennard–Jones (LJ) potentials with a cutoff radius of 1 nm are applied to model vdw interactions within the PCFF framework. The velocity-Verlet integration algorithm is utilized to solve Newton’s equations of motion, with a time step set to 1 femtosecond (fs) to ensure computational accuracy and stability.

**Figure 1:**
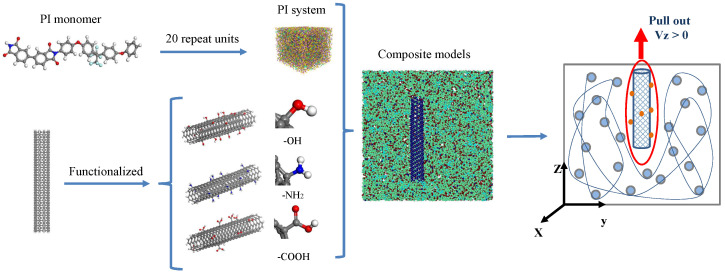
Schematic diagram of composites models of CNT/PI nanocomposites.

The widely used two-component system, consisting of biphenyltetracarboxylic dianhydride (BPDA) and a hexafluoropropane-containing aminophenoxy diamine, is selected for the PI matrix. The chemical structure and atomic model of a single segment are shown in Figure 1. For the nanofiller, an armchair (5,5) SWCNT with a diameter of 6.78 Å and a length of 49.19 Å is chosen, as previous studies indicate that CNT diameter and chirality have limited effects on the Young’s modulus. Although the CNT length and corresponding aspect ratio (i.e., 7.2) are smaller than those in experiments, this model can reproduce sufficient load transfer at the interface while preventing a rapid drop in stress near the ends of the CNT. Aromatic polyimide molecular dynamics simulations are computationally demanding due to the long chain length and the presence of rigid cyclic motifs (e.g., benzene and imide rings). Models with many repeat units require prohibitively long simulation times and substantial memory, whereas excessively short chains may introduce finite-size effects. Although short-chain models cannot fully reproduce the complete conformational behavior of real polymer chains, numerous studies have reported reasonable results for polyimide systems using chains containing 10–20 repeat units [32,33]. In the modeling process, the nanocomposite structure is initiated by inserting the SWCNT fragment into the simulation cell and randomly generating 20 polyimide chains, each with 20 repeat units. It should be noted that the present simulations model the solid-state CNT/PI interface without explicit solvent molecules. While solvent effects may influence CNT dispersion and interfacial formation during composite processing, the focus of this study is on the interfacial mechanical properties of the cured composite, in which solvent is absent. This simplification is consistent with the approach adopted in the majority of MD studies on CNT/polymer interfacial mechanics in the literature.

To systematically investigate the impact of functional groups on the interfacial properties of functionalized CNT/PI nanocomposites, three types of functionalized CNTs are synthesized to construct interfacial CNT/PI models, as illustrated in Figure 1. These functional groups include hydroxyl (-OH), amine (-${\mathrm{NH}}_{2}$), and carboxyl (-COOH). The functional groups were covalently bonded to randomly selected carbon atoms on the CNT sidewall through C–C single bonds, with the interatomic distances automatically satisfying the geometric requirements of the PCFF force field. To further elucidate the influence of functional group content on interfacial behavior, each type of functional group is randomly grafted onto the surface of CNTs at varying concentrations of 2.5%, 5.0%, 7.5%, and 10.0%. The content of surface-modified functional groups on CNTs is defined as the ratio of the number of functional groups to the number of carbon atoms on the CNT surface.

To eliminate structural residual stress and achieve a reasonable density, the system is relaxed through an annealing process within 300-600 K for 10 times in 2.5 ps (∆t=0.25fs) and 4 times in 320 ps (∆t=1fs). This is followed by a series of dynamic equilibrium simulations including 200 ps at 300 K and 1 atm in the isothermal-isobaric (NPT) ensemble, 200 ps at 300 K in the canonical (NVT) ensemble, and 200 ps in the microcanonical ensemble (NVE). The resulting polymer system has side lengths of 72.8974 Å × 67.6259 Å × 81.1763 Å. The simulation system is then heated to 820 K and relaxed for 0.5 ps (∆t=1fs) under the NPT ensemble to obtain a well-relaxed initial structure. Subsequently, the temperature is decreased stepwise by 20 K increments down to 460 K with 0.5 ns of dynamics simulation at each temperature under the NPT ensemble. It should be noted that all functionalized CNT/PI systems underwent identical annealing and equilibration protocols, ensuring that the observed differences in interfacial properties arise solely from variations in functional group type and content, rather than from differences in thermal history or initial configurations.

### 2.2. Simulation Methods

The nanocomposites structures are equilibrated under the isothermal conditions at 300 K for 0.1 ns following initial energy minimization. Prior to the loading process, the edges of the polyimide matrix are constrained to prevent simultaneous drift with the CNT along the loading direction. An additional 1 ns of equilibration at 300 K is performed to further relax the constrained system. ISS of the nanocomposite systems is determined using the steered molecular dynamics (SMD) approach through pull-out simulations. Inspired by atomic force microscopy (AFM) techniques, SMD simulations are employed to provide microscopic insights into the mechanical response and molecular mechanisms governing interfacial behavior. Similar to the pull-out experiments using an AFM tip, SMD simulation is conducted by applying an external force to the end of CNT in constant velocity mode. Each atom within the defined loading region of the CNT is subjected to a force of magnitude $K\left[vt-(R-R_{0})\right]\frac{m_{i}}{m}$, where *K* is the spring constant, *v* is the pulling rate, $R_{0}$ is the initial equilibrium position of the center of mass (COM) of the loading region and R(t) is the position of the COM of the loading region at time *t*, $m_{i}$ is the mass of the atom, and *m* is the total mass of the CNT. During the pull-out process, the CNT is extracted along the axial direction (z axis) at a constant velocity of $1\times 10^{-4}$ Å/fs, driven by a steered force generated by tethering a spring to one end of the CNT. Due to the limited time scale in MD simulations, the selected pulling velocity is several orders of magnitude higher than that used in the AFM experiments. However, this approach effectively captures the interfacial behavior between the embedded filler and the surrounding matrix without influence from molecular thermal vibrations. The work done during the pulling process is averaged over multiple independent configurations using Jarzynski’s equality along the pulling path, providing an estimate of the potential of mean force (PMF) as the pull-out energy. The average ISS can be estimated based on the pull-out energy $E_{po}$, as indicated in Equation (1):

(1) $$\tau =\frac{2E_{po}}{\pi DL^{2}}$$

where *D* and *L* are the diameter and the embedded length of the CNT, respectively.

## 3. Results and Discussion

### 3.1. Effect of Functionalization on Interfacial Energy

**Figure 2:**
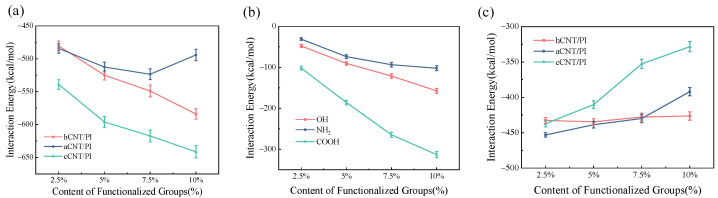
Interaction energy between PI and (**a**) CNT ($E_{t}$), (**b**) functional groups ($E_{fc}$), and (**c**) CNT surface excluding the functional groups ($E_{pc}$).

To systematically investigate the influence of functional group types and concentrations on the interfacial shear properties between PI and CNTs, the interfacial strength of the CNT/PI system is first evaluated, as it fundamentally governs the interfacial shear behavior. As shown in Figure 2a, we calculate the interaction energy of PI matrices modified with different functional groups (-OH, -${\mathrm{NH}}_{2}$, and -COOH) at CNT surface modification densities ranging from 2.5% to 10%. The total interaction energy between Carbon nanotubes and the polyimide matrix is denoted as $E_{t}$. $E_{fc}$ represents the interaction energy between PI and the functional groups, while $E_{pc}$ signifies the interaction energy between PI and pristine CNTs. The relationship among these energies can be expressed by the following equation:

(2) $$E_{t}=E_{fc}+E_{pc}$$

For clear identification of the samples, functionalized CNT are systematically labeled using a nomenclature system in which “nCNT” denotes a CNT modified with a specific functional group, where “n” represents the first letter of the group name. Specifically, hCNT, aCNT, and cCNT correspond to CNTs functionalized with -OH, -${\mathrm{NH}}_{2}$, and -COOH groups, respectively.

As shown in Figure 2a, regardless of the functional group content, the ranking of the interfacial interaction energy among the three CNT/PI systems—cCNT/PI, hCNT/PI, and aCNT/PI—remained consistently cCNT/PI>hCNT/PI>aCNT/PI. The observed order of cohesive energy for the functional groups aligns with that reported by Trolier-McKinstry et al. [34], exhibiting a decreasing trend as follows: -COOH> -OH> -‘${\mathrm{NH}}_{2}$’. These results indicate that CNTs functionalized with higher-polarity groups (such as -COOH or -OH) exhibit stronger interfacial binding energy compared to those functionalized with lower-polarity groups (such as -${\mathrm{NH}}_{2}$).

Moreover, as the functional group content increased from 2.5% to 10.0%, the interaction energies became more negative, indicating stronger CNT–PI interactions; the magnitudes of the interaction energies increased by 19.0% for cCNT/PI and 21.5% for hCNT/PI (relative to the 2.5% case). Conversely, the change in the interfacial energy of the aCNT/PI system is negligible. Notably, during the calculation of the interaction energy between PI and the CNT functionalized with the -${\mathrm{NH}}_{2}$ group, the interfacial interaction energy does not show a positive correlation with the number of interfacial interacting atoms, failing to reflect the actual contribution of the functional group to the interfacial binding energy. Consequently, the interaction energies between PI and each functional group are calculated and compared, as illustrated in Figure 2b. As anticipated, the magnitudes of the interaction energies between the PI matrix and the functional groups increase as the functional group content rose from 2.5% to 10%. Specifically, the increases are 229.3% for the PI-OH group model, 229.2% for the PI-${\mathrm{NH}}_{2}$ group model, and 207.5% for the PI-COOH group model. This outcome is attributed to the increased number of functional group atoms, which enhances the likelihood of interactions between the functional groups and the PI matrix. This preliminarily suggests that increasing the number of functional groups on the CNT surface can significantly boost the interaction energy of the CNT/PI system. Furthermore, given that the order of the interaction energies between the PI matrix and the functional groups are consistent with that between the PI matrix and the CNT at a given content, we further postulate that the interaction energy between the two components may be influenced by both the polarity and size of the functional groups.

However, it is observed that the slope of the curve representing the change in interaction energy decreases as the functional group content increases. For instance, in the case of the PI/-OH group system, as the content of -OH functional groups on the CNT increases from 2.5% to 10%, the slopes of the curve are 0.8898, 0.3405, and 0.2997, respectively. This suggests that the interfacial interaction between the -OH groups and the PI matrix approaches a saturation state with increasing functional group content. A similar conclusion can be drawn by comparing the other two functional groups. Additionally, when comparing the interaction energies $E_{pc}$ between PI and the CNT surface (excluding the functional groups), the interaction energies of the three functionalized samples gradually decreased with the increase in the number of functional groups, as shown in Figure 2c. Among these, the sample with -COOH exhibited the largest decrease. This phenomenon can be attributed to the inhibition of PI matrix adsorption on the CNT surface, resulting from the excluded-volume effect. This effect arises from non-bonded interactions between the functional groups on the CNT surface and the PI matrix, which are influenced by the content and size of the functional groups. This indicates that over-functionalization introduces a competing negative effect: while the covalently attached functional groups enhance hydrogen bonding with the PI matrix, the steric exclusion effect simultaneously reduces the van der Waals interaction between the PI chains and the CNT carbon backbone, partially offsetting the gains in interfacial strength.

**Figure 3:**
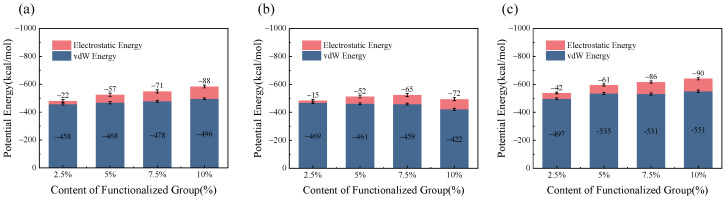
The contribution of electrostatic energy and vdw energy to the interfacial interaction energy of the (**a**) hCNT/PI system, (**b**) aCNT/PI system, and (**c**) cCNT/PI system.

Through statistical analysis of the interfacial interaction energies in CNT/PI nanocomposite systems as a function of functionalization type and content, the results show that the interfacial bonding between PI and CNTs is primarily governed by the polarity of the functional groups. However, the interfacial interaction energies consist of the sum of vdw interactions and Coulomb interactions, which also include the vdw potential energy between PI and CNT. The influence of the polarity of functional groups on the interfacial bonding properties cannot be evaluated solely through interfacial interaction energy. Therefore, in this study, the interaction energy is numerically calculated by combining the vdw energy and electrostatic energy between the CNT and the PI matrix. Figure 3 shows the contributions of vdw potential energy and electrostatic potential energy to the interfacial interaction energy, revealing that vdw potential energy makes a greater contribution to the interaction between PI and CNT. This primarily originates from the interaction between PI and the electrically neutral surface of CNT. However, by analyzing Figure 3b,c, it can be observed that when the content of functional groups increases from 5% to 7.5%, the vdw interaction potential between PI and CNT decreases. This indicates that the modification with polar functional groups contributes little to the vdw interaction between CNT and PI; instead, it causes a reduction in the vdw interaction due to the excluded volume effect. Additionally, under the condition of the same number of functional groups, the electrostatic interaction potential in systems with higher-polarity functional groups is greater than in those with lower-polarity groups. For example, at 7.5% content, the electrostatic interaction potentials of the cCNT/PI and hCNT/PI systems are 85.9 kcal/mol and 70.51 kcal/mol, respectively, while that of the aCNT/PI system is 64.5 kcal/mol. Through comparison, it is found that the proportion of the electrostatic interaction potential in the total interfacial interaction energy is higher in the cCNT/PI and hCNT/PI systems, which contain functional groups with higher polarity, than in the aCNT/PI system. This indicates that the differences in the polarity of functional groups are reflected in the electrostatic interaction potential. The electrostatic potential of functional groups is primarily influenced by the electronegativity of polar atoms and the distribution of force-field charges. The strong electronegativity of polar atoms also increases the likelihood of hydrogen bonds (H-bonds) formation, which is one of the main reasons for the increase in interfacial energy.

As shown in Figure 3a–c, the interfacial interaction in the cCNT/PI system is stronger than that in the hCNT/PI system at the same functional group content; however, the difference in electrostatic potential between the two is not significant. For example, at a 10% content, the electrostatic potential values between hCNT and PI, and between cCNT and PI are 87.52 kcal/mol and 90.03 kcal/mol, respectively. This is because the -COOH functional group contains two polar groups (-OH and -C=O), both capable of forming H-bonds. However, due to the close proximity of these groups, they cannot provide sufficient contact sites to form H-bonds with PI, thereby reducing the probability of hydrogen bond formation between PI and the -COOH functional group. To validate these hypotheses, it is essential to investigate the effects of functional group type and content on H-bonds formation to understand the underlying mechanism.

### 3.2. Effect of Functionalization on Hydrogen Bonds

**Figure 4:**
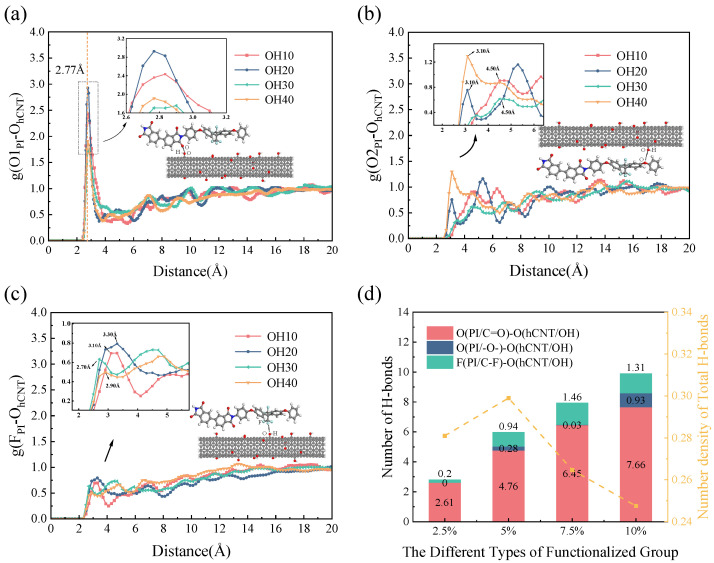
RDF plot of (**a**) O atom of the -C=O group in PI and O atom of the -OH group in hCNT and (**b**) O atom of the -O- group in PI and O atom of the -OH group in hCNT and (**c**) F atom of the -F- group in PI and O atom of the -OH group in hCNT (**d**) Number of H-bonds in PI/hCNT.

**Figure 5:**
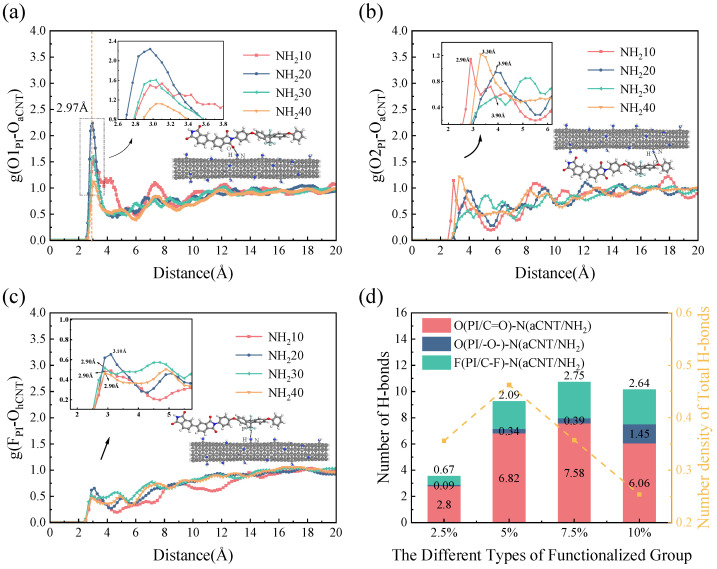
RDF plot of (**a**) O atom of the -C=O group in PI and O atom of the -${\mathrm{NH}}_{2}$ group in aCNT and (**b**) O atom of the -O- group in PI and O atom of the -${\mathrm{NH}}_{2}$ group in aCNT and (**c**) F atom of the -F- group in PI and O atom of the -${\mathrm{NH}}_{2}$ group in aCNT (**d**) Number of H-bonds in PI/aCNT.

**Figure 6:**
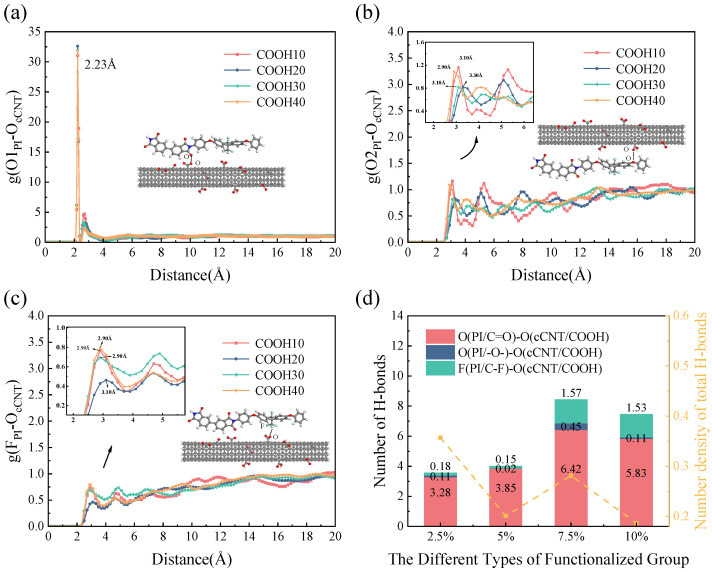
RDF plot of (**a**) O atom of the -C=O group in PI and O atom of the -COOH group in cCNT and (**b**) O atom of the -O- group in PI and O atom of the -COOH group in cCNT and (**c**) F atom of the -F- group in PI and O atom of the -COOH.

While prior literature has indicated that the interfacial binding strength between CNT and PI can be influenced by the polarity of functional groups through H-bonds interactions, few studies have explicitly reported the donor–acceptor relationships of H-bonds between the PI matrix and CNT. Additionally, although some reports have noted that increasing the functional group content can enhance interfacial strength, the underlying mechanisms and the impact of content level on interfacial strength remain unclear. Therefore, to qualitatively investigate the effect of the type and content of functional groups on the formation of H-bonds between CNT and PI, as well as to clarify the nature of H-bonds between PI and CNT, the radial distribution function (RDF) and the number density of H-bonds are introduced. In the following discussion, $O1_{\mathrm{PI}}$ denotes the oxygen atom of the carbonyl (-C=O) group, $O2_{\mathrm{PI}}$ represents the oxygen atom of the ether (-O-) group, and $O_{F}$ indicates the fluorine atom in the PI backbone. The formula for calculating the RDF is

(3) $$g\left(r\right)=\frac{1}{\rho 4\pi r^{2}\delta r}t=\frac{\sum_{t=1}^{T}\sum_{j=1}^{N}∆N\left(r\to r+dr\right)}{N\times T}$$

where ρ is the system density (quantity density), *N* is the total number of atoms, *T* is the computation time (steps), and *r* is the radius from the reference atom. According to previous literature, H-bonds are identified based on the criteria that the donor–hydrogen-acceptor angle exceeds 150° and the distance between the donor and acceptor is less than 3.5 Å. However, the types and polarities of hydrogen atoms as donors or acceptors can affect the geometric configuration of H-bonds, thereby influencing their bond strength. This indicates that clarifying the types of formed H-bonds is crucial when analyzing the effect of hydrogen bonding on interfacial binding properties. Therefore, it is necessary to conduct research on the formation of H-bonds between CNT modified with different functional groups and PI. In calculating the RDF, the oxygen atoms in the C=O, -O-, and -F groups of the polymer are designated as H-bond acceptors, while the -OH and -NH_2_ groups on the CNT surface serve as H-bond donors (through their O-H and N-H bonds, respectively), while the oxygen and fluorine atoms in the C=O, -O-, and -F groups of PI serve as acceptors. Taking the -OH group as an example, the RDF of the functional groups C=O, -O-, and -F with -OH are denoted as g($O1_{\mathrm{PI}}$-$O_{\mathrm{hCNT}}$), g($O2_{\mathrm{PI}}$-$O_{\mathrm{hCNT}}$), and g($O_{F}$-$O_{\mathrm{hCNT}}$), respectively. Figure 4, Figure 5 and Figure 6 illustrates the RDF of H-bonds donors and acceptors potentially forming H-bonds between PI and CNTs functionalized with -OH, -${\mathrm{NH}}_{2}$, and -COOH groups, respectively. In Figure 4, Figure 5 and Figure 6, the first peak of each curve represents the average distance between the selected polar atoms in the equilibrium structure, while the curve height indicates the degree of atomic aggregation. To effectively compare the H-bonds formed by different functional group contents, the H-bonds generated by varying numbers of functional groups are normalized and defined as the H-bond density. The H-bond density, denoted as $\rho_{h}$, is defined as the ratio of the number of H-bonds formed (i.e., $N_{h}$) to the total number of functional groups (i.e., $N_{f}$) at a given functional group content, as expressed by Equation (4):

(4) $$\rho_{h}=\frac{N_{h}}{N_{f}}$$

As shown in Figure 4, the first peak in the RDF of ($O1_{\mathrm{PI}}$/$O_{\mathrm{hCNT}}$, $O2_{\mathrm{PI}}$/$O_{\mathrm{hCNT}}$ and $F_{\mathrm{PI}}$/$O_{\mathrm{hCNT}}$ in hCNT/PI nanocomposites appears at 2.77 Å, 3.1 Å and 2.7 Å, respectively. Peaks within 3.5 Å in the RDF indicate the presence of covalent or hydrogen bonds between two atoms, while peaks beyond 3.5 Å suggest vdw interactions and Coulomb forces. It is believed that $O1_{\mathrm{PI}}$/$O_{\mathrm{hCNT}}$, $O2_{\mathrm{PI}}$/$O_{\mathrm{hCNT}}$, and $F_{\mathrm{PI}}$/$O_{\mathrm{hCNT}}$ can all form H-bonds. Additionally, compared to the RDF of $O2_{\mathrm{PI}}$/$O_{\mathrm{hCNT}}$ and $F_{\mathrm{PI}}$/$O_{\mathrm{hCNT}}$, a sharper peak at the initial position is observed in the RDF of $O1_{\mathrm{PI}}$/$O_{\mathrm{hCNT}}$. These results indicate that the donor-acceptor distance between $O_{\mathrm{hCNT}}$ and $O1_{\mathrm{PI}}$ is smaller than the distances between $O2_{\mathrm{PI}}$/$O_{\mathrm{hCNT}}$, $F_{\mathrm{PI}}$/$O_{\mathrm{hCNT}}$ and $O_{\mathrm{hCNT}}$. This suggests that the probability of H-bond formation is highest between $O_{\mathrm{hCNT}}$ of the -OH groups on the surface of CNT and $O1_{\mathrm{PI}}$ of the C=O groups on the PI chains. This finding is also reflected in the difference in the number of these two types of hydrogen bonds, as shown in Figure 4d. As shown in Figure 4d, the number of hydrogen bonds formed between OhCNT and $O1_{\mathrm{PI}}$ is greater than that between $O2_{\mathrm{PI}}$, $F_{\mathrm{PI}}$, and $O_{\mathrm{hCNT}}$, indicating that the first type of hydrogen bond plays a major role in the binding energy. As observed in Figure 4a, with the increase in the content of -OH functional groups, the peak value of the first peak of the g(r) curve does not exhibit a monotonically increasing trend with the increase in functional groups. Instead, a maximum value appeared at 5%, indicating that at this concentration, the attractive force between the oxygen atoms of the -OH groups on hCNT and the C=O groups on PI at the interface of the hCNT/PI system reaches its maximum, making it impossible to adsorb more functional groups. This also suggests that the O–H···O type H-bonds formed between the -OH donor on hCNT and the C=O acceptor of PI.

To further analyze the influence of functional group content on interfacial H-bond formation and to verify the accuracy of RDF results, the number of interfacial H-bonds is statistically analyzed based on established H-bonds identification criteria, as shown in Figure 4d. The figure reveals that as the content of functional groups modified on the CNT surface increases, the number of H-bonds formed at the hCNT/PI interface gradually rises. However, the H-bond density reaches a maximum when the functional group content is 5%. This plateau is attributed to the randomness of functional group modification on the CNT surface and the volume exclusion effect induced by the functional groups. These findings indicate the existence of a content threshold in the process of interfacial H-bonds formation. Beyond this threshold, no additional H-bonds can form at the CNT/PI interface, thereby limiting further enhancement of the interfacial bonding strength.

For the aCNT/PI model (i.e., Figure 5), the first peak of the RDF between $O1_{\mathrm{PI}}$ in the C=O group and $N_{\mathrm{aCNT}}$ in the -${\mathrm{NH}}_{2}$ group appears at 2.97 Å. Meanwhile, the peaks of the RDF between $O2_{\mathrm{PI}}$ in the -O- group, $F_{\mathrm{PI}}$ in the -F- group and $N_{\mathrm{aCNT}}$ all emerge at 2.90 Å. This indicates that N-H⋯O type H-bonds can form between $N_{\mathrm{aCNT}}$ and $O1_{\mathrm{PI}}$, $O2_{\mathrm{PI}}$, and $F_{\mathrm{PI}}$. However, the peak value of the g($O1_{\mathrm{PI}}$-$N_{\mathrm{aCNT}}$) curve is higher than those of the g($O2_{\mathrm{PI}}$-$N_{\mathrm{aCNT}}$) and g($F_{\mathrm{PI}}$-$N_{\mathrm{aCNT}}$) curves. Thus, $O1_{\mathrm{PI}}$ of the C=O group has the highest probability of forming H-bonds. As observed in Figure 5a, as the content of the -${\mathrm{NH}}_{2}$ functional groups increases, the peak value of the first peak of the g(r) curve reaches a maximum when the functional group content is 5%, suggesting that this type of H-bond has reached saturation at this point. In contrast, as the functional group content increases, the position of the single peak of $O2_{\mathrm{PI}}$ in the -O- group shifts to 3.9 Å, which exceeds the previously established cutoff distance for H-bonds. This implies that at the functional group contents of 5% and 7.5%, the polar atom pair $O2_{\mathrm{PI}}$-$N_{\mathrm{aCNT}}$ between the PI molecules and the aCNT system cannot form H-bonds (as shown in Figure 5b). The peak value of the RDF between $F_{\mathrm{PI}}$ of the -F- group and $N_{\mathrm{aCNT}}$ of the -${\mathrm{NH}}_{2}$ group reaches a maximum at the 2.5%, however, the overall g(r) value does not exceed 0.75. In general, by comparing the position of g(r) and the first peak, it can be concluded that the strength of the H-bond between $O1_{\mathrm{PI}}$ and $N_{\mathrm{aCNT}}$ is greater than that between $O2_{\mathrm{PI}}$, $F_{\mathrm{PI}}$, and $N_{\mathrm{aCNT}}$. This result is also reflected in the difference in the number of H-bonds, as shown in Figure 5d. The number of H-bonds formed by $O1_{\mathrm{PI}}$-$N_{\mathrm{aCNT}}$ is significantly greater than those formed by $O2_{\mathrm{PI}}$-$N_{\mathrm{aCNT}}$ and $F_{\mathrm{PI}}$-$N_{\mathrm{aCNT}}$, with the H-bond density reaching a maximum at 5% content.

For the cCNT/PI model (i.e., Figure 6), the peak values of the RDF curves for $O2_{\mathrm{PI}}$-$O_{\mathrm{cCNT}}$ and $F_{\mathrm{PI}}$-$O_{\mathrm{cCNT}}$ are significantly lower than that for the $O1_{\mathrm{PI}}$-$O_{\mathrm{cCNT}}$ pair. This indicates a much weaker interfacial interaction. As shown in Figure 6d, even at a functional group content of 10%, the number of H-bonds formed in the $O2_{\mathrm{PI}}$-$O_{\mathrm{cCNT}}$ and $F_{\mathrm{PI}}$-$O_{\mathrm{cCNT}}$ pairs does not exceed 1 and 2, respectively, suggesting a negligible probability of H-bond formation. In contrast, the RDF curve for $O1_{\mathrm{PI}}$-$O_{\mathrm{cCNT}}$ in Figure 6a exhibits a sharp, distinct peak at 2.23 Å. This peak corresponds to the formation of an O–H···O type H-bond, where the -OH group of the -COOH functional group on the cCNT acts as the H-bond donor, and the C=O group of the PI molecule acts as the acceptor. The hydrogen donation ability of the -OH group is significantly enhanced by the adjacent electron-withdrawing carbonyl (C=O) group within the -COOH functionality. In addition, the carbonyl group in the -COOH functionality acts as a H-bond acceptor, forming weak interactions with the C=O groups of PI and thereby increasing the number of available binding sites. This combination of donor and acceptor groups creates a synergistic effect, which collectively contributes to a significant enhancement of the PI-cCNT interfacial interaction.

Furthermore, a comparison of Figure 4d and Figure 6d reveals that the hCNT/PI system, which relies on O–H···O hydrogen bonding, requires a higher functional group content to reach interfacial saturation than the cCNT system. This difference is attributed to the molecular structure of the -COOH group. In addition to the -OH donor, the carboxyl group possesses a carbonyl (C=O) moiety, which introduces greater steric hindrance. This heightened steric exclusion in the cCNT/PI system reduces the number of accessible contact sites between the carbonyl groups of PI and the -OH donors on the CNT, thereby lowering the probability of forming O–H···O H-bonds and leading to an earlier saturation.

This H-bond saturation, combined with the monotonic decrease in $E_{pc}$ driven by the excluded-volume effect described in Section 3.1, collectively demonstrates that over-functionalization introduces competing negative effects on the CNT/PI interfacial interaction. To further validate the hydrogen bond analysis presented above, the interfacial potential energy decomposition results in Section 3.1 (Figure 3) are revisited. Since hydrogen bonds are composed of both electrostatic and van der Waals interactions, with electrostatic interactions being the dominant component, systems with a higher hydrogen bond density are expected to exhibit larger electrostatic interaction energies. As shown in Figure 3, the electrostatic interaction energies follow the order cCNT/PI > hCNT/PI > aCNT/PI, which is consistent with the hydrogen bond density results obtained from the RDF analysis and hydrogen bond counting in this section. This agreement between the energy decomposition and the geometric hydrogen bond characterization provides mutual validation of the two analyses.

### 3.3. Effect of Functionalization on Shear Properties

**Figure 7:**
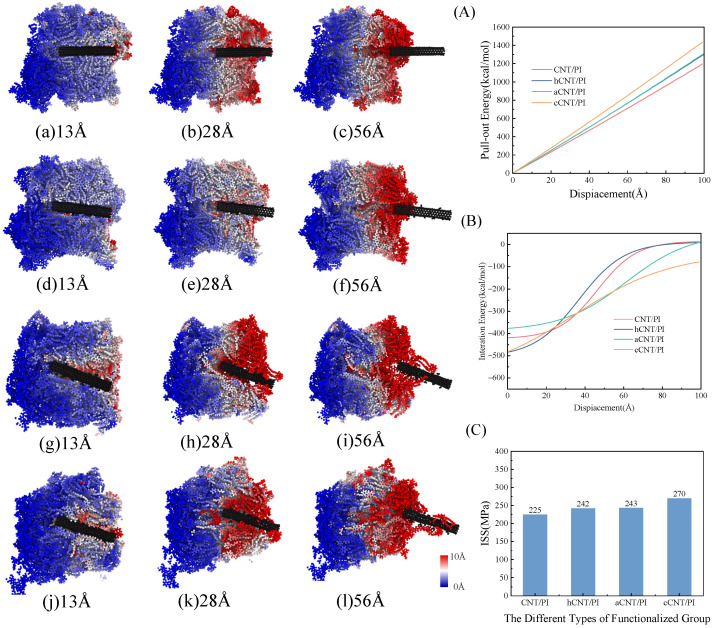
Pull-out simulation results for pristine and functionalized CNT/PI systems. (**a**–**l**) Cross-sectional views showing atomic displacement during pull-out at different stages for CNT/PI, hCNT/PI, aCNT/PI, and cCNT/PI systems. (**A**) Pull-out energy vs. displacement. (**B**) CNT-PI interaction energy vs. displacement. (**C**) Interfacial shear strength comparison for different functionalized CNTs.

To investigate the interfacial properties and load transfer capability between the CNT and the PI matrix, we perform pull-out simulations on both pristine and functionalized CNT/PI nanocomposites. The interactions between the CNT wall and the polymer matrix are governed by non-bonded interactions, primarily van der Waals forces and hydrogen bonding. The PMF obtained from SMD simulations is plotted against displacement in Figure 7A. The results indicate that the pull-out work increases approximately linearly with displacement, with a higher value for the functionalized CNT than for the pristine CNT. Furthermore, the CNT-PI interaction energy, plotted in Figure 7B, exhibits a negative value, confirming an attractive interaction between the CNT and the PI matrix. The simulation results confirm that functionalization significantly enhances the interfacial interaction between the CNT and the PI matrix. This improvement is quantitatively reflected in the ISS, which was calculated from the pull-out energies using Equation (1). The ISS of the pristine CNT–PI interface is calculated as 225 MPa. By comparison, the interfaces between polymer matrix and functionalized CNTs presented higher ISS values, varying from 242 MPa to 269 MPa. The obtained results are of the same order of magnitude as previously reported MD simulation values in the literature, including ISS values of 100–300 MPa for carbon fiber/polyimide composites [35] and 400–500 MPa for amino-functionalized CNT/epoxy composites [36], demonstrating the reasonableness of the present model. The maximum observed improvement of approximately 20%, achieved in the cCNT/PI system, demonstrates that appropriate functional group selection effectively promotes stronger interfacial adhesion. Since ISS directly governs the efficiency of stress transfer from the matrix to the CNT, this enhancement is expected to translate into measurable improvements in the macroscopic mechanical performance of the composite, including tensile strength and fracture toughness.

To further explore the load transfer mechanism in the nanocomposites, the relative atomic displacements are estimated between the adjacent atomic configurations. The interactions between the CNT and PI matrix cause the local deformation of nearby polymer chains during the pull-out process. In the beginning for the pristine CNT case, only the PI chains at the adjacent region around the interface show large displacements as shown in Figure 7a. In the case of functionalized CNT, the PI chains surrounding the CNTs are affected by the movement of the functional groups, as shown in Figure 7d,g,j. The pull-out of CNT in the nanocomposites releases the preoccupied space inside the PI matrix, which allows the relaxation of PI chains at the free end of CNT. The PI chains start to move towards the vacancy surrounding the tail of CNT, and such movement is captured in both nanocomposite systems in Figure 7b,e,h,k. The atoms of PI matrix are more active in the functionalized CNT case, suggesting more PI chains are affected by the functional groups. Before the complete pull-out, the regions with larger displacements are mostly located in the pull-out direction of the CNT, and the amount of these atoms is greater in the functionalized CNT case than that in the pristine CNT case as compared in Figure 7c,f,i,l. The functionalized CNT interacts with more PI chains, leading to more severe local deformation during the pull-out process and higher ISS in CNT-PI nanocomposite. The results suggest that the introduction of the functional groups can cause better mechanical interlocking in nanocomposites, which effectively strengthens the interface between CNT and PI matrix.

## 4. Conclusions

This study employs MD simulations to investigate the reinforcement of PI nanocomposites by CNTs functionalized with different types and contents of functional groups. By analyzing the interfacial energy, RDF, number of H-bonds and H-bond number density, we elucidate the relationship between interfacial bonding strength and the hydrogen bonding interactions. The results indicate that CNTs modified with -COOH groups most effectively enhance the interfacial bonding energy, which is attributed to stronger electrostatic and H-bonding interactions conferred by the high polarity of the carboxyl group. While the number of H-bonds tends to saturate with increasing functional group content, pull-out simulations reveal a significant improvement in the ISS for functionalized CNTs. Specifically, the cCNT exhibit a 20% increase in ISS compared to the pristine CNT. The stronger interfacial interactions and the more significant local deformation observed during the pull-out process in functionalized systems facilitate more efficient load transfer between the CNT and the PI matrix. It should be noted that the present findings are based on armchair (5,5) SWCNT, and the influence of CNT chirality and diameter on the CNT-PI interfacial behavior warrants further investigation. This work provides microscopic insights into the interfacial mechanics of functionalized CNT/PI nanocomposites, offering valuable guidance for the design of advanced polymeric materials.

## Data Availability

The original contributions presented in this study are included in the article. Further inquiries can be directed to the corresponding author.
